# The Telenovela Effect: Challenges of Location Filming and Telenovela Tourism in the Brazilian Favelas

**DOI:** 10.1111/jpcu.12861

**Published:** 2020-01-02

**Authors:** Débora Póvoa, Stijn Reijnders, Emiel Martens

On January 5, 2018, an article in *The Washington Post* noted the rising violence in Rocinha, the largest favela in Rio de Janeiro, Brazil. With the headline “A Once‐Trendy Rio Slum Is Now ‘at War,’” it detailed how the “showcase shantytown in Brazil's showcase city” had become a conflict zone, with a fourfold increase in killings in less than a year (Faiola and Kaiser). The first favela in Rio to receive tourists, Rocinha had recently gathered media attention for drug gangs that (re)started gunfights there. In October 2017, the slaying of a tourist by the police further destabilized its reputation as the prime destination of favela tourism in Rio. As journalist Misha Glenny explains, “if there is trouble in Rocinha, there is a guarantee of even greater problems elsewhere.” Indeed, violence had also stricken other communities in the city, causing a sharp decrease in tourism, according to various Brazilian news reports (Brito; A. Mendonça; Ouchana and Galdo).

These recent events seem to jeopardize a long period of prosperity in favela tourism in Brazil. In the past twenty‐five years or so, the favelas in Rio—and, to a lesser extent, in São Paulo—have become among the most iconic images of the country, together with the triad of carnival, soccer, and beaches (Williams 487). Once considered a social and sanitary problem by Rio de Janeiro's government (Valladares 12‐13), these territories have gone through a process of rebranding largely set in motion and sustained by the convergence between tourism and media. By propagating a shared representation of the favelas as exotic and dangerous, these two industries have transformed them into global commodities, which are now consumed by filmmakers (as locations) and tourists (as attractions).

However, becoming a popular brand often comes at a price. In *Community Development through Tourism*, Sue Beeton states that sometimes “communities are left to face the results of a too successful … image with limited resources and understanding” (137). Beeton has shown that host communities targeted for tourism potentially face problems of misrepresentation and exploitation (122‐23, 191‐216). In the particular case of slum communities, such as the favelas, literature on locals’ perception of tourism remains scarce (Diekmann and Chowdary 113; Freire‐Medeiros, “Listening to Local Voices” 175‐76; Slikker and Koens 76). In addition, very few studies have dealt with the impact of the filming process on locations and the respective local opinion (see, e.g., Brydon and Stead 101‐14).

As a response to this research gap, this article analyzes the use of the favelas Complexo do Alemão in Rio de Janeiro and Paraisópolis in São Paulo as filming locations and tourist attractions through the eyes of residents who are either directly or indirectly involved in these uses. These two communities were respectively depicted in the Brazilian telenovelas *Salve Jorge* (writ. Glória Perez, dir. Marcos Schechtman and Fred Mayrink, 2012–13) and *I Love Paraisópolis* (writ. Alcides Nogueira and Mario Teixeira, dir. Carlos Araújo and Wolf Maya, 2015)—both produced and broadcast by the Brazilian television company Rede Globo. The two telenovelas became rather popular and soon provoked tourists to visit their locations. However, these tourism flows did not last very long. Tourism decreased in both communities, just as it had before in Rocinha and other favelas in Rio de Janeiro. In light of this scenario, how local residents of Complexo do Alemão and Paraisópolis perceive the use of their communities as filming locations and tourist attractions is worth analysis. Through this line of inquiry, we propose the *telenovela effect*: the scope of the telenovelas’ impacts (including their instigation of tourism) on location communities before, during, and after their releases. In this study, interviews with twenty individuals^1^ involved in media and/or tourism in their respective favelas reveal that, although generally seen as a way to improve the living conditions of these communities, the telenovela effect ends up being ephemeral. This ephemerality is due to the equally transient nature of the telenovela genre and the challenges these territories experience in terms of security and governmental investment.

## Narratives and Uses of the Favelas in Media and Tourism

Since the first slum community was settled in Rio de Janeiro at the end of the nineteenth century, the favelas have had contradictory associations in the Brazilian and global popular imaginary. On the one hand, they are seen as zones of high criminality and violence, and on the other they are considered the birthplace of typically Brazilian cultural expressions. As Beatriz Jaguaribe points out, “celebratory versions of the favela as a samba community composing carnival lyrics coexist with images of armed adolescents shooting police forces during drug raids” (327). In Brazil's national public debate, however, it is the negative stereotype that has often prevailed, with the favelas being mostly stigmatized and neglected by the country's state and business sectors. These communities and their residents have been, and still are, targets of eradication campaigns and are confronted with police brutality and civil rights infringements on a daily basis (Livingstone 20; Prouse 8). Social exclusion also remains a problem, since “the very stigma of living in a designated ‘non‐place’ confers low status, exclusion, mal‐treatment, and derision, independently of a person's assets, livelihood or overall income” (Perlman 28). In 2018, Brazilian president Jair Bolsonaro promised that, once elected, he would give the police carte blanche to kill during gunfights with alleged criminals (Benites; Pinheiro‐Machado), which implies that civilian casualties from police fire could also go unpunished. In a city like Rio de Janeiro, where the death of innocent people, including children, in police raids is already an alarming issue (Morte de inocentes), such a promise added to the state of fear and susceptibility in which favela populations live.

While these communities and their residents are shunned by Brazilian politicians and wealthy elites, they are hailed in other meaning‐making practices, namely media and tourism. Since 1992, when the first group of tourists visited Rocinha, the Brazilian favelas have been incorporated in the rising global phenomenon of slum tourism, which includes other poverty‐stricken areas such as Mumbai, Kibera, and South Africa's townships (Frenzel and Koens 196‐98). In this type of tourism, poverty—often resignified into narratives of diligence, cultural uniqueness, solidarity, among others—is the main commodity exchanged between tour operators and tourists (Frenzel and Koens 199). Analyzing favela tourism in particular, Bianca Freire‐Medeiros argues that it combines elements of both social and dark tourisms: Tourists are allowed an altruistic engagement with favela communities while being motivated to consume the adverse conditions that these places experience (“Touristic Transits” 582). Alongside tourism, media productions have also used the favelas for entertainment purposes. One example is when singer Michael Jackson released the music video “They Don't Care About Us” in 1996, which used the favela Santa Marta as one of its settings.

The connections between the media and tourism industries are evident in film tourism: “the visitation to sites where movies and TV programs have been filmed as well as to tours to production studios, including film‐related theme parks” (Beeton, *Film‐Induced Tourism* 11). The favelas are a case in point: The Oscar‐nominated *City of God* (dir. Fernando Meirelles and Kátia Lund, 2002) has been regarded as largely responsible for the establishment of these places as popular tourist spots in Rio (Freire‐Medeiros, “Touristic Transits” 582). The film depicts the community of Cidade de Deus as “an artistically creative, convivial, and vital favela coexisting with the hell of the drug trade” (Peixoto 173). It presents an image of the favelas appealing to tourists: poverty and risk on the one hand, and solidarity and cultural richness on the other (Freire‐Medeiros, “A Construção da Favela Carioca”). With this, cinematic representations and tourism practices in the favelas mutually shape the global imagination of them. According to Freire‐Medeiros, “if the touristic favela carries the burdens of displaced representations proposed by *City of God*, the cinematic favela is also shaped by the vocabularies of tourism and the demands of potential travelers” (“City of God” 25).

Since *City of God*, convergent cinematic and tourist narratives about the favelas have proliferated, allowing for a “travelling favela” to emerge: “a space of imagination and a mobile entity that is travelled to while travelling around the world” (Freire‐Medeiros, *Touring Poverty* 4). More than a shared spatial imaginary, the favelas have become a global trademark capable of hyping up any leisure enterprise, from hostels and restaurants to bars and parties. Devoid of any geographical marker or cultural specificity, the word “favela” in itself has been appropriated by, for example, an Asian restaurant in Sydney, Australia, and a techno club in Münster, Germany (Freire‐Medeiros, “Touristic Transits” 583), becoming an exotic brand to any entertainment business, anywhere on the globe.

More recently, the favelas have also been used as a backdrop for Brazilian telenovelas. Timidly introduced into the genre in the 2000s, these neighborhoods have gained prominence as main settings of the telenovelas *Salve Jorge*,* I Love Paraisópolis*,* Babilônia* (2015) and *A Regra do Jogo* (2015‐16). In Brazil, telenovelas are usually exhibited six times per week on free‐to‐air television, with three productions consecutively airing in standard time slots—at 6 p.m., 7 p.m., and 8 or 9 p.m. Their production rhythm is industrial, with a clear and segmented work division (directors, writers, costume, set designers, etc.) and a strategic planning in terms of costs and target audience (Oguri et al. 41‐42). Also, their writing and filming occur simultaneously to their exhibition. At the date of their release, there are only twelve to eighteen pre‐filmed episodes to be exhibited in the first weeks. Afterward, six new episodes are produced and broadcast per week (Oguri et al. 42). This allows for the authors to change elements of the story depending on the audience's reaction (Silva 152): If a telenovela has low ratings, its plot, for example, can be altered or its duration shortened (Oguri et al. 43). Once a telenovela is over—generally after an average of 160 episodes (Silva 169)—it is immediately replaced by another in the allocated time slot. These characteristics attest to the accelerated production rhythm of the genre and its profit‐driven nature. Besides, they make the case for the ephemerality and replaceability of these productions: With such pervasiveness on Brazilian television, only a few of them enjoy long‐lasting popularity.

In the midst of so many narratives about and uses of the favelas, the opinion of favela populations about such practices deserves attention. The few studies on favela tourism that include residents’ perspectives reveal that most of them are either indifferent or positive about tourism practices taking place in their backyards (Diekmann and Chowdhary 124; Freire‐Medeiros, “Listening” 181; Kieti and Magio 54; Slikker and Koens 12). However, the use of the favelas as filming locations and tourist attractions can take its toll on favela communities. Often led by outsiders, that is, people who do not live in, or belong to, the favela, both media and tourism industries frequently operate within the framework of exoticism. According to Graham Huggan, exoticism converts cultural difference into terms that can be easily understood by a mainstream audience (22)—in this case, television and movie viewers and tourists—often resorting to stereotypical discourses about certain places and peoples (201‐02). In the case of the favelas, these discourses are caught in fascination and primitivism: stereotypes that have created the “travelling favela” and also historically contributed to the stigmatization of these territories and their residents.

The discourses about the favelas affect the practices of location filming, media viewing, and tourism, which can also be detrimental. Throughout the years, favela tourism has often been considered a reflection of the power imbalance between the visitor and the resident, with the latter potentially being subjected to invasions of privacy and disrespect (Whyte et al. 345‐46). Case studies on media tourism have shown that local communities can see film shootings as a nuisance because they disturb daily routines by, for example, creating traffic congestion and street restrictions (Bolan et al. 246). Other research has suggested that media tourism may not be sustainable. Glen Croy argues that films have the potential to economically contribute to a place during the production phase, but long‐term economic gains through post‐production effects, like tourism, are harder to measure (161). Only a small number of films actually generate tourism flows, and even these flows might not be significant (160). Considering their frequent turnover and the fast pace of their production, the telenovelas’ sustainability is arguably even more fragile since they can be more easily forgettable—and thus less likely to motivate tourism to their filming locations.

In order to understand if that was indeed the case, twenty interviews were conducted with residents of Complexo do Alemão and Paraisópolis involved in media and/or tourism in their respective favelas—including tour^2^ guides,^3^ local entrepreneurs, and community leaders. They were asked how they were dealing with, and what they expected from, film producers and tourism entrepreneurs who use their neighborhoods as film locations or tourist attractions, focusing on their experiences with the telenovelas *Salve Jorge* and *I Love Paraisópolis*. It is important to note, however, that due to the relatively limited sample of interviewees, the conclusions drawn from this study cannot be generalized to the entirety of these favelas’ populations. However, we believe that the findings provide valuable insights about the workings of location filming and telenovela tourism in the Brazilian favelas—and potentially in other marginalized urban areas around the globe.

## Filming in the Community

The interviews in Paraisópolis reveal that local residents who were involved in the production of *I Love Paraisópolis* are more likely to praise the telenovela's contribution to the community than those who were not. The location filming of *I Love Paraisópolis* lasted three months. During pre‐production visits to the community, the Rede Globo network negotiated with the Residents’ Union, headed by Gilson Rodrigues at the time. After obtaining the Union's approval, the telenovela's directors and producers started doing research in the favela to create the characters, as Gilson explained: “There was a long period of research here, I think more than a year, [a period] of people circulating here, looking, knowing, experiencing, really sitting, and having a beer, listening to the local accents.” According to Gilson, during the actual shooting of the telenovela, Rede Globo hired more than a hundred locals to work as extras or as support staff.

Two of these freelance workers, Renata 2017, 2017 and Higor 2017, 2017, were interviewed for this study. They helped the production crew understand the atmosphere of the neighborhood, the way residents dress and behave, and the history of the favela. Some locals even became the inspiration for characters of the telenovela, including Renata herself, who would also talk directly to the actors to correct them in case they were not accurately mimicking the local accent or slang. Therefore, Renata, Higor, and Gilson were offered the opportunity to voice their own perceptions about Paraisópolis and to bring the telenovela as close to their reality as possible, in an attempt to make themselves and other residents feel represented.

However, residents who were only indirectly involved in the filming process often did not notice this effort. For example, the community leader José Maria Oliveira complained about the lack of local participation: “I wanted a telenovela … of the real life. [For the production to] arrive at a party, film the people dancing. Get two, three dancers to be featured in the scene, even for one second. Then it would be a telenovela from the neighborhood. But I didn't see any of this.” His criticism was repeated by other interviewees, who complained about the lack of locals’ perspective as well as the limited number of scenes actually filmed in the community. The vast employment of locals, although confirmed by Gilson and Renata, was not perceived by other residents, who mentioned that Globo only hired a few as extras and barely filmed in the community itself. This minimal (perceived) interaction and collaboration with the residents, although welcome, was not considered enough, as the community leader Betânia Mendonça explained:

Everybody thought that the telenovela was filmed here. And they shot four, five scenes here, tops. They assembled a whole set at Projac [Rede Globo's production studios in Rio de Janeiro] … and they filmed everything there. … So that's what I'm talking about. [It] only depleted us, because they took away all our richness [and] made money out of things we don't even know.

Gilson confirmed that Globo filmed in the community until it had the telenovela set ready at Projac.^4^ Even though the production crew wanted to shoot more scenes in the community, it became difficult to manage the residents logistically. As Renata recalled:

At some point it became untenable to shoot here, we would waste [a lot of time] … Because it would gather a lot of people. Shooting was impossible. And there was that thing of “guys, five minutes of silence, don't take pictures now.” When we would start filming someone would take their cell phone out. Tcha, tcha, tcha, tcha! [imitating the sound of flashes] … And then we had to start all over again.

The above hints at a discrepancy in profile and power between the Residents’ Union and some residents who were not directly involved in the filming negotiation and execution. For residents from sensitive areas, such as the favelas, the location filming of a media production is often seen as an opportunity to improve their living conditions. For example, the community leader William 2017, 2017 stated that he “wanted Rede Gobo to create a studio here in Paraisópolis … because it would bring more employment for Paraisópolis, more culture … In this condition of crisis that we're living … it would bring assets for the community.” In reality, however, the opportunity is often there for a selected few and only for a short time span, primarily the narrow window in which the production is being made. In this sense, the ephemerality and selectivity of the benefits that the filming of the telenovela brought to Paraisópolis are related to the strict nature of the making of the genre. The need for an efficient filming schedule in which disruptions have to be minimized and the filming on location cannot become too costly restrains a broader collaboration with the community.

In the case of Complexo do Alemão, local people interviewed for this study by and large appreciated the short‐term benefits of the location shooting of *Salve Jorge*. Most respondents were positive about the participation of residents, mentioning that Rede Globo hired extras, security personnel, and caterers from the community. The local entrepreneur Mariluce Mariá Souza appreciated the temporary jobs the filming of the telenovela created for the unemployed: “They didn't really hire, they made daily payments to the person. [Yet] this was positive because even if you are unemployed it puts the workforce here in the favela in motion.” Two interviewees, though, argued that more local workforce could have been hired.

As with some respondents in Paraisópolis, the residents of Complexo do Alemão noticed that very few scenes of the telenovela were filmed on location. As the tour guide André Valle recalled: “Most [of the scenes] were [done in] a set. They might've filmed only 10% of Alemão … [Filming] here was only to give legitimacy [to the telenovela].” The limited filming on location also had an impact on the tourist experience. As the local tour guide Cristiano Ferreira explained: “The favela itself was in Projac … There was a famous bar in the telenovela. [The tourists] wanted to know where it was, and I had to say, ‘that's only in the telenovela.’ The streets and alleys you see [in the telenovela] don't exist here.” However, the residents did not see the reduced number of scenes in the favela as a problem, but were rather indifferent or even positive about it. After all, less filming in the community means less disturbances for the community. Of more concern to the interviewees was the representation of Complexo do Alemão in the telenovela *Salve Jorge*, which for most of them became another “outsider” depiction of the favela.

## Favela Representation in the Telenovelas

Misrepresentation in the telenovelas was an issue raised by many respondents in both Complexo do Alemão and Paraisópolis. They generally criticized the inaccuracy of characters, whom they deemed stereotypical. According to Jessica Souto, a former tour guide from Complexo do Alemão, “it ends up being the same old thing of someone from the outside coming, doing their thing and [claiming] ‘ah, this is about that favela.’ In the end a lot of people don't feel represented.” The tour guide André Valle argued that *Salve Jorge* did not show the different types of people of the favela:

Usually the characters that Globo puts in its plots are very stereotypical, right? … The woman is a tease, the guy is a swindler … [There's] the drunk guy, the smart youngster who likes [Brazilian] funk and can become a thug … See? And they don't create a character of a Black guy who is attending university and dreams of becoming a lawyer. There are plenty of them here. Plenty.

Even when showing characters based on real‐life residents or situations that actually took place in the favelas, the telenovelas have often been blamed for romanticizing them. Jessica mentioned that the way the telenovela portrayed the Pacification^5^ process in Complexo do Alemão concealed the moments of terror experienced by the residents: “It was very much like ‘ah, such a heroic act, very beautiful,’ and ‘look, how nice, [the leading lady] fell in love with a policeman to save the favela.’ … [In reality] people were hiding from gunshots … Everybody [was] scared.” Whereas people in Complexo do Alemão complained about the glorification of the police, in Paraisópolis, the community leader Gilson suggested that drug dealers were deified: The character Grego, supposedly a criminal and the “owner” of Paraisópolis in the telenovela, was in love with the leading lady and ended up being portrayed as a “good guy.” The problem with this strategy, however, is that it gives the impression that being a drug dealer is not so bad. As Gilson explained:

in a moment [in Brazil] when there is much talk about ostentation, about power, and when there are no good role models in the country, … you have a guy [in the telenovela] who is a drug dealer, … showing his wealth and having by his side a very beautiful and desired woman. And you have that [shown] to a population where 35% of the people are youngsters between fifteen‐ and twenty‐nine‐years old.

In response to these critiques, Renata, who participated in the composition of the characters of *I Love Paraisópolis*, said that many of them had to be “toned down” from their more realistic traits to suit the telenovela's early time slot (7 p.m.). For example, the writers could only implicitly suggest that Grego was a drug dealer. For Renata, this softer characterization was intended to avoid denigrating the favela's image on TV.

In spite of these complaints about representation, both in Complexo do Alemão and Paraisópolis, the respondents at times also pointed out some positive outcomes of the TV productions. Jessica said that, although generally showing Complexo do Alemão in a very superficial way, the telenovela was also rather playful in dealing with the local scenery:

When a telenovela … shows that in the favela there are moments of conflict, but there's also the guy at the bar who jokes around, who throws a party every now and then, that there are boys flying kites, the girls [sunbathing] on the rooftops, or the very nice people who live there, this automatically stimulates people to ask themselves if the favela is really that bad thing in the newspaper, or if it's really a spot to be explored like other parts of the city.

Moreover, according to some respondents, the telenovelas gave at least some positive visibility to the favelas. According to the president of the Women's Association of Paraisópolis, Elizandra 2018, 2017, *I Love Paraisópolis* challenged the common media representation of the community, especially in the news: “You get a lot more audience if you show in your newscast that there is a gunfight here … than if you show that we have a ballet school … that we have girls being considered for [the Russian ballet company] Bolshoi … [Crime] unfortunately still draws more attention.” According to Elizandra, then, even though the way the telenovela depicted Paraisópolis was still not ideal—in her opinion, the plot became too comic and, again, some characters too unrealistic—it was better than usual.

This resonates with what several other interviewees argued. The telenovelas seemed to bring positive affirmations of pride and self‐esteem among residents. As the community leader Gilson 2018, 2017 explained: “Our parents had to say they lived in Morumbi [an upper class neighborhood next to Paraisópolis] for them to get an opportunity. … Nowadays, if you say ‘I'm from Paraisópolis,’ people say ‘I Love Paraisópolis.’ So this is a concrete change that the telenovela brought.” A similar shift happened in Complexo do Alemão. As former tour guide Paulo 2017, 2017 related: “I got in the job market only after this culture started to be shown [in the telenovela and tourism] … because [before], when we said we lived in Alemão, no one gave us job opportunities. We were discriminated against.” In other words, people who previously would refrain from saying they lived in Complexo do Alemão or Paraisópolis started to disclose that information with pride when their respective telenovela aired. However, while in Paraisópolis this renewed self‐esteem has perdured. According to some interviewees in Complexo do Alemão, the positive outcomes lasted for only a limited period of time. As former local tour guide Anderson Lima recounted: “When the telenovela started, the resident of Alemão would be proud. He would say, ‘I live in Complexo do Alemão.’ … This until 2014 [the year of the return of conflicts between drug gangs and policemen in the community]. After that everything started again.”

In the interviewees’ view, the telenovelas never seemed to find an appropriate balance in the representation of Complexo do Alemão and Paraisópolis. While at times they repeated long‐standing stereotypes about the favelas, they also overly attenuated certain aspects of these places because of content restrictions related to the telenovelas’ timeslots. However, this flawed representation was still better than not being represented at all. The visibility given by the telenovelas to these communities renovated the sense of pride and belonging of their residents, even though, in Complexo do Alemão, the limited endurance of *Salve Jorge*—179 episodes spanning seven months—and the instability of the favelas, in terms of security, prevented this effect from being long‐lasting.

## After‐Release Effects

From the interviews it also appeared that, once the telenovelas were broadcast, they generated a sudden yet equally temporary interest in the favelas. According to former local tour guide Paulo 2017, 2017, in Complexo do Alemão more projects and business opportunities emerged during the broadcasting period: “When the telenovela was airing, … there was everything here. Events, a lot of events … [However,] when the telenovela ended, it was all over. … Now only the ones that really care about the community remained.” As for Paraisópolis, the interviewees who directly worked in the production of the telenovela mentioned that the attention that the community received by the media at the time—not only the telenovela itself, but also news reports and interviews with locals—gave more visibility to local entrepreneurs, which temporarily boosted their businesses. Artisan Berbela^6^ was one of the people who benefitted from this ephemeral interest: “Through the telenovela, I became more well‐known, people got excited and curious to come and see the [art] pieces, and [business] improved a lot. … When the telenovela was over it decreased a bit.” Renata also noticed that there was more demand for filming in the favela: “The fact that Higor and I actively participated [in the making of the telenovela] opened this door for other productions to look for us.” Other residents, though, did not notice any significant positive change. Community leader William Bastos mentioned that “Paraisópolis remained the same.”

In terms of tourism, all respondents in Complexo do Alemão agreed that the telenovela *Salve Jorge* momentarily boosted the tourism that already existed in the community. The production inspired the creation of specific tours and souvenirs and was mentioned by tour guides, depending on the tourists’ interests. In Paraisópolis, however, the answers were more divided, with some interviewees noticing a temporary increase in tourists and others not perceiving any impact whatsoever.

Part of the explanation of why the influence of the telenovela was either considered temporary or barely noticed in these favelas is that these productions were not the main factors for the rise of tourism in the first place. In Complexo do Alemão, the construction of a cable car^7^ was referred to as the main reason, with the telenovela reportedly being of secondary importance. As Jessica Souto indicates,

The main point is the cable car. The cable car started to feature in newspaper headlines as a tourist attraction … But then that's it, in the period of the telenovela the big public [for tourism] was because of the telenovela. But then even after the telenovela ended, over time, the public that came talking about the telenovela gradually diminished, so [the reason] was more the cable car indeed.

In Paraisópolis, interviewees cited the presence of local artisans and qualified tour guides, the urbanization of Paraisópolis, and the spread of the fame of favela tours in Rio to São Paulo as the main reasons tourism occurred. The telenovela itself was never mentioned. For the outsider tour operator Luis Simardi, the demand existed before the telenovela: “I suspect that if there was an impact it was momentary … because I see that there was not any new company doing [the tour to Paraisópolis] … You see that there was a demand already in place, I think that [the telenovela] didn't increment [tourism] that much.”

Nowadays, the influx of tourists is very small in both communities. When asked about what made tourism decrease again, respondents in Complexo do Alemão said it was the rise of violence, especially due to police operations, and the closing of the cable car. The end of the telenovela was considered important only because it coincided with other events—the beginning of the low tourist season, the upsurge in violence, and the increase of negative reports about the community in the media. In Paraisópolis, the end of the telenovela was never mentioned as a factor for the decrease of tourism. Outsider tour guides said that the increase of violence was the main problem. However, Gilson affirmed that the cultural circuit offered by the Residents’ Union continues to receive the same, albeit “not so expressive” number of visitors as always.

In general, the respondents were skeptical about the capacity of media productions to sustain tourism in their communities. When asked about what is needed for tourism to be successful again in Complexo do Alemão, interviewees mentioned the reopening of the cable car and the solving of the security issues that the community faces nowadays. In Paraisópolis, the need for more infrastructure both for tourists and locals—hotels, but more importantly, sanitation and paved streets—was considered the main factor. As Jessica sums up:

There is potential here, but it doesn't depend … on a media strategy. I think Alemão has issues that go beyond this media strategy. While this security issue is not solved or partially defined, I think that everything remains too unstable. So even if the strategy is good and even if it works in terms of audience impact, I think it won't be long‐lasting.

Thus, any after‐effects of the telenovelas were deemed largely temporary. Investors apparently lost interest in these neighborhoods once the hype created by the TV productions lost momentum, and tourism entrepreneurs faced challenges that went beyond the popularity of the telenovelas—namely the closing of main tourist attractions and, most importantly, the rise of violence. Hence, trusting in media productions as a means of development in the favelas on their own is unfeasible. The limited period of exhibition of the telenovelas, together with the historical and structural issues that these communities experience, hinder the telenovelas’ power to sustain a positive image of the favelas that could potentially generate new business and tourism opportunities for their residents.

## Conclusion: The Telenovela Effect

According to the analysis of twenty interviews conducted in the two communities, we can say that in areas that have faced stigmatization and neglect for so long, media productions and related tourism practices are seen by locals as an opportunity for development, self‐expression, and empowerment—but they often fail to fully live up to these expectations.

As our case studies have shown, most residents interviewed for this research wanted the telenovela crews to work together with the community in the production phase, to provide a more realistic representation of these neighborhoods during the broadcast period, and to leave a long‐lasting imprint in the favelas once the telenovelas ended. Ultimately, however, the outcome of the filming of the telenovelas was largely considered ephemeral: a temporary collaboration, a temporary pride, and a temporary boost in business and tourism.

One of the explanations for this is the telenovela genre itself. Its fast‐paced, cost‐effective style of production does not allow for an extensive filming period on location, restraining the possibilities of collaboration with the community. The timeslots of the telenovelas also restrict the type of content they can show, which resulted in the residents interviewed by this study deeming *I Love Paraisópolis* and *Salve Jorge* inaccurate: by toning down characters and repeating stereotypes, for example. Besides, the limited airing period of the telenovelas translated into an equally limited popularity and sense of self‐esteem, especially in Complexo do Alemão. In the end, the *telenovela effect*, that is, the scope of the telenovelas’ impacts before, during, and after their releases, was *like* a telenovela: short‐lived and fast‐paced. Thus, beyond the specific outcomes observed in this study, the term *telenovela effect* can potentially also be used to refer to the ephemerality of the effects that telenovelas—and arguably other media forms—have on their filming locations.

Hypothetically, though, the memory of a telenovela could still be preserved on the filming location—and continue to generate income for locals through tourism—as long as references to it are created. These might include the production of souvenirs (as done in Complexo do Alemão) and the heavy marketing of telenovela tours. However, this research has shown that such an endeavor might be unfeasible in the favelas due to the instability that these sensitive urban areas face: limited financial resources, waves of violence, and other adversities derived for the most part from governmental neglect. The second reason for the ephemerality of the telenovela effect, then, can be ascribed to the locations themselves. The favelas, and arguably other less advantaged territories, offer scholars and policymakers alike complexities and specificities for location filming and tourism that go beyond a successful media production. With this, we claim that the potential often attributed to media tourism in privileged contexts is not entirely applicable to areas such as favelas. As part of a larger project, this study is the first step toward a better understanding of the complexities of these territories. Further research can be conducted—in other sensitive contexts and with other media and tourism practitioners, that is, film commissions and governmental bodies—to provide a broader panorama of media tourism in less privileged areas.

## Notes

1 The communities of Paraisópolis in São Paulo and Complexo do Alemão in Rio de Janeiro were visited by the first author two times, once in 2017 and once in 2018. Semi structured, in‐depth interviews were conducted with people who were recently engaged, in diverse ways, in the dynamics of location filming and favela (media) tourism: ten (former) tour guides, six community leaders, that is, people associated with communitarian organizations such as Residents’ Unions, and four local entrepreneurs. The interviews were carried out either one‐on‐one or in small groups ranging from two to four people. In the process of interviewing, the first author tried to understand the practice of location filming and related favela tourism from the subjects’ perceptions. This allowed us to understand the conflicting interests and power relations between local residents on the one hand, and media and tourism entrepreneurs on the other.

2 We use the term “tour” to broadly designate any guided excursion in the favelas, even though in Paraisópolis the local Residents’ Union calls their initiative a “cultural circuit.” This distinction will be made only when we refer to community‐based excursions in Paraisópolis.

3 We use the term “tour guide” to designate all people who conducted tourism in these communities, regardless of them being accredited by the Ministry of Tourism (and therefore professionalized) or not. Moreover, while in Complexo do Alemão the first author spoke only with local tour guides, in Paraisópolis she also interviewed three outsiders. This is due to the fact that all community‐based excursions in this favela are managed by the local Residents’ Union. Therefore, by including outsider tour guides, she was able to gather multiple opinions about favela tourism and its relationship with the telenovela.

4 Projac, currently called Globo Studios, was created in 1995 to increase productivity and reduce production costs of Rede Globo's TV programs, among other reasons (Oguri et al. 41).

5 Starting in 2008, the Pacification of Rio's favelas consisted of the implementation of Pacifying Police Units (UPPs), first in the favela Santa Marta and subsequently in another thirty‐seven communities. The project was created to contain the violence in the favelas in the run‐up to the 2014 World Cup and the 2016 Olympic Games (2015, 2017 179). Nowadays, with the return of violent conflicts between policemen and drug dealers, the Pacification is considered a mostly failed initiative (2018, 2017).

6 Berbela primarily works as a mechanic, but he became famous in Paraisópolis for making sculptures out of car engine pieces. He was hired by Rede Globo to build the sculptures that were featured in the opening sequence of the telenovela, besides also appearing in its final episodes and having a character partially inspired by him.

7 Inaugurated in 2011, the cable car in Complexo do Alemão carried 9,000 people daily. It was closed in 2016, in principle due to an atypical damage in one of the cables. Now, Rio de Janeiro's state government claims that there are not sufficient funds for it to start functioning again (Ribeiro).
